# Using Robots at Home to Support Patients With Chronic Obstructive Pulmonary Disease: Pilot Randomized Controlled Trial

**DOI:** 10.2196/jmir.8640

**Published:** 2018-02-13

**Authors:** Elizabeth Broadbent, Jeff Garrett, Nicola Jepsen, Vickie Li Ogilvie, Ho Seok Ahn, Hayley Robinson, Kathryn Peri, Ngaire Kerse, Paul Rouse, Avinesh Pillai, Bruce MacDonald

**Affiliations:** ^1^ Department of Psychological Medicine Faculty of Medical and Health Sciences The University of Auckland Auckland New Zealand; ^2^ Department of Respiratory Medicine Counties Manukau District Health Board Auckland New Zealand; ^3^ Acute Allied Health Department Counties Manukau District Health Board Auckland New Zealand; ^4^ Department of Electrical and Computer Engineering The University of Auckland Auckland New Zealand; ^5^ School of Nursing Faculty of Medical and Health Sciences The University of Auckland Auckland New Zealand; ^6^ School of Population Health Faculty of Medical and Health Sciences The University of Auckland Auckland New Zealand; ^7^ Department of Accounting and Finance The University of Auckland Auckland New Zealand; ^8^ Department of Statistics The University of Auckland Auckland New Zealand

**Keywords:** robotics, chronic obstructive pulmonary disease, hospitalization, medication adherence, telehealth, rehabilitation exercise

## Abstract

**Background:**

Socially assistive robots are being developed for patients to help manage chronic health conditions such as chronic obstructive pulmonary disease (COPD). Adherence to medication and availability of rehabilitation are suboptimal in this patient group, which increases the risk of hospitalization.

**Objective:**

This pilot study aimed to investigate the effectiveness of a robot delivering telehealth care to increase adherence to medication and home rehabilitation, improve quality of life, and reduce hospital readmission compared with a standard care control group.

**Methods:**

At discharge from hospital for a COPD admission, 60 patients were randomized to receive a robot at home for 4 months or to a control group. Number of hospitalization days for respiratory admissions over the 4-month study period was the primary outcome. Medication adherence, frequency of rehabilitation exercise, and quality of life were also assessed. Implementation interviews as well as benefit-cost analysis were conducted.

**Results:**

Intention-to-treat and per protocol analyses showed no significant differences in the number of respiratory-related hospitalizations between groups. The intervention group was more adherent to their long-acting inhalers (mean number of prescribed puffs taken per day=48.5%) than the control group (mean 29.5%, *P*=.03, *d*=0.68) assessed via electronic recording. Self-reported adherence was also higher in the intervention group after controlling for covariates (*P*=.04). The intervention group increased their rehabilitation exercise frequency compared with the control group (mean difference −4.53, 95% CI −7.16 to −1.92). There were no significant differences in quality of life. Of the 25 patients who had the robot, 19 had favorable attitudes.

**Conclusions:**

This pilot study suggests that a homecare robot can improve adherence to medication and increase exercise. Further research is needed with a larger sample size to further investigate effects on hospitalizations after improvements are made to the robots. The robots could be especially useful for patients struggling with adherence.

**Trial Registration:**

Australian New Zealand Clinical Trials Registry ACTRN12615000259549; http://www.anzctr.org.au (Archived by WebCite at  http://www.webcitation.org/6whIjptLS)

## Introduction

### Background

Chronic obstructive pulmonary disease (COPD) is a progressive respiratory disease that primarily involves airflow obstruction. The worldwide prevalence of COPD is around 10% in men and 6% in women, and it is projected to be the third leading cause of death by 2020 [1]. As the disease progresses, the frequency and severity of exacerbations increase. On average, patients are thought to experience around 3 exacerbations per year, with approximately 50% of these going unreported and untreated.

Treatment for COPD primarily involves medication and behavioral changes such as smoking cessation and exercise. Pulmonary rehabilitation (consisting of exercise, education, and support) can improve patient outcomes and reduce hospital admissions and health care costs [1]. Patients with moderate, severe, and very severe COPD benefit more from rehabilitation than patients with mild forms of the disease, and home-based programs are as effective as hospital-based programs [2]. Despite its benefits, the availability and uptake of rehabilitation programs are low [3,4].

There are several risk factors for COPD-related hospital admissions, including nonadherence to medication [5]. Medication adherence in COPD patients is around 50% and has been linked with better education, higher satisfaction with the patient-provider relationship, and less depression [6]. Hospital admissions for COPD exacerbations are expensive, with the average cost per admission in New Zealand (NZ) being around NZ $4800 [7]. More research is needed to work out ways to improve adherence and make home-based rehabilitation available for people with moderate to severe COPD.

Technological innovations, such as telephone-based interventions, are being increasingly used to help patients with chronic conditions manage their condition. However, there is little evidence for the effectiveness of many telehealth interventions. Although a review of 9 small studies of varying quality found that home telephone support and telemonitoring could reduce the rate of hospitalization and emergency department visits in COPD patients and increase patient satisfaction and quality of life, the review recommended larger higher-quality trials to be conducted [8]. A meta-analysis of 80 systematic reviews of telehealth care (3 of which were with COPD) found that mortality for COPD patients was not affected but hospitalizations were reduced and quality of life was increased; however, this review also recommended larger high-quality studies to be performed [9]. A large study of Home Telehealth tested with veterans in the United States reduced bed days of care by 25% and hospital admissions by 19%, but this was not a randomized trial [10]. Larger and more recent randomized trials have found that telehealth has no effects on hospitalizations or health-related quality of life in COPD patients compared with standard care, and more research is needed to identify subgroups who are most likely to benefit and into mechanisms behind any effects [11,12].

A new and emerging form of technology for delivering remote care is the socially assistive robot. Assistive robots are generally acceptable to people, and there is some evidence that they can improve loneliness and quality of life [13-16] and reduce costs in rural medical practice [17,18]. Due to their social presence, robots may engage people more with health interventions than other telehealth configurations. There is preliminary evidence that people have increased adherence to instructions from a robot than from a computer delivering health instructions [19,20]. However, there are no long-term randomized controlled trials investigating whether robots can improve adherence to medication and reduce hospitalizations in patient groups.

### Aims

The aims of this pilot study were to provide data on the feasibility, clinical effectiveness, and cost-effectiveness of a robot delivering telehealth care to patients with COPD. The study was not powered to show effects. Older patients, people from rural areas, and Māori and Pacific peoples were targeted for recruitment in this study, because risk of readmission is higher for these groups [7]. This paper presents the primary outcome—that is, hospitalization—along with a cost-effectiveness analysis and the secondary outcomes of medication adherence and quality of life. The hypotheses were that the robot would reduce days of hospitalization, increase adherence, increase exercise, and improve quality of life compared with a control group.

## Methods

### Trial Design

A parallel randomized controlled trial was conducted. Patients were randomized using a 1:1 allocation ratio and stratified based on ethnicity and gender. The statistician (AP) generated the random number sequence using a randomization program and kept this in a separate location to the research team. The physiotherapists (NJ and VLO) recruited participants in hospital, and after collection of the baseline questionnaires, emailed the statistician with the gender and ethnicity of the participant to find out his/her group allocation. Participants were randomized to either receive a robot in their homes for 4 months in addition to standard care or to receive standard care alone. The physiotherapists informed each participant of their group allocation. Participants could not be blinded to group allocation.

### Ethics, Consent, and Permissions

Approval was obtained from the New Zealand Health and Disability Ethics Committees (Ref 14/NTA/229) and from Counties Manukau District Health Board Research Review Committee. Written informed consent was obtained from all participants.

### Participants

Participants were recruited face to face from Middlemore Hospital (Counties Manukau District Health Board) at the time of discharge. Inclusion criteria were as follows: confirmed diagnosis of COPD, COPD-related admission, previous admission in past year, gets out of the house less than 4 times per week, living alone or with spouse who is also largely housebound, geographic rural location, poor social support, and aged between 16 and 90 years. All patients recruited into the study had preexisting lung function tests available in their medical record. All participants had a forced expiratory volume in 1 second (FEV_1_)/forced vital capacity (FVC) ratio of <0.7, and 37% were classified as severe (30%<FEV_1_<50% predicted) and 50% as very severe (FEV_1_<30% predicted).

Exclusion criteria included elevated levels of NT-pro BNP (N-terminal pro b-type natriuretic peptide) and Troponin T, CURB-65 score>2 (CURB, *C* onfusion, blood *U* rea nitrogen>19 mg/dL, *R* espiration rate >30, *B* lood pressure<90 systolic blood pressure or <60 diastolic blood pressure, age >65) [21], on long-term oxygen therapy at time of admission (all of which predict an increased risk of dying within 30 days), incurable cancer, residence in rest home, and working. The study originally planned a 6-month intervention period and for recruitment to occur at 2 locations, but the period was shortened to 4 months and recruitment occurred at only one location because of logistical difficulties and a limited funding period. After consent was obtained, participants completed baseline questionnaires in hospital. The trial protocol can be accessed by emailing the corresponding author.

### Intervention

The iRobi robot (Yujin Robot Limited, Korea) has previously been used for health care in retirement and rural settings in New Zealand [18,22]. For this trial, it was programmed to deliver COPD management consisting of several components guided by a clinical pathway. The overall program was designed to monitor health and prompt medical contact if health was deteriorating.

The detailed functions were as follows: (1) measure pulse oximetry, forced expiration volume, heart rate, and symptoms, mental state, and functional status using the Clinical COPD Questionnaire (CCQ) on a weekly basis [23]; (2) remind patients when to take medication and inhalers and record their adherence several times a day; (3) remind patients to do their rehabilitation exercises and display videos of a patient performing these at least twice weekly; (4) provide education about COPD via video modules and pop-up messages; (5) allow participants to use an *I am feeling unwell* function on demand; and (6) show trends over time of health status and adherence on its screen to the patient. The robot was integrated with Wi-Fi–linked Smartinhalers (Adherium, New Zealand) to monitor inhaler use. The data were sent to a secure Web server that managed all robot and patient data and logged all activities, with alerts if the measurements were out of range or patients were not adherent.

When the *I am feeling unwell* button was pressed, the robot asked whether or not the situation was an emergency. If yes, then the robot advised the participant to dial 111 (emergency services). If no, then the robot asked whether their current health concern was related to their COPD. If yes, then the robot initiated assessment of pulse oximetry, forced expiration volume, heart rate, and CCQ and sent the study physiotherapist an SMS text message (short message service [SMS]) alert. Otherwise, the robot advised the participant to contact their general practitioner. Patients were advised that if the *I am feeling unwell* assessment was completed outside work hours, then the physiotherapist would call the participant on the next working day. Patients were informed that they should carry on with the usual ways that they dealt with health problems outside work hours because the robot server was only monitored 5 days a week from 9 AM to 5 PM. The alert function was also triggered if any parameters were outside the normal range, if medications were missed more than 3 consecutive times (either on Smartinhaler data or robot server), or if exercise was missed more than 3 consecutive times.

Two part-time physiotherapists were employed to monitor data via a Web browser and were asked to carry a cell phone to answer calls or respond to alerts. They checked the server every working day. If the physiotherapists detected an adherence or health-related issue, they phoned the patient to discuss concerns and passed on relevant information to respiratory physicians if necessary. If patients were not using the robots, they were encouraged to do so in the phone-call. Blood tests for biomarkers were arranged in some cases and treatment was adjusted where indicated. Patients were encouraged to see their general practitioner rather than the hospital team. The modules were designed by the multidisciplinary team (robotic engineers, psychologist, nurse, general practitioner, medical student, respiratory physician, and physiotherapists). Although patients were not involved in the design of the modules, our team has previous experience in designing such modules for other health conditions, and we did test the robot with a few patients before the study began to gather feedback and check its usability. The programmed scenarios included handling of out-of-range health data, emergency conditions, and input errors.

The physiotherapists delivered the robots and Smartinhalers to the homes of the participants in the robot group approximately 1 week after discharge. During the interim week, the engineers set up each robot with the associated Smartinhalers and Internet connection devices for each individual. The physiotherapists introduced the purpose of the robot as helping the patients to comanage their condition with the medical care team. They explained that patients should use the robot every day and explained what the robot did, why, and when, and demonstrated all the functions. They also explained that the Smartinhalers tracked the patient’s inhaler use, and thus, the physiotherapists could monitor if the participants were using the inhalers enough or too much. Patients were given a written manual with instructions on how to use the robot and Smartinhalers. Internet connectivity for the robot was set up by the researchers, and Internet costs were covered by the study.

### Control Group

The control group received standard care (usual care from the general practitioner, hospital inpatient and outpatient services, and rehabilitation program). This included referral for rehabilitation and contact with respiratory physiotherapists and other clinicians as needed. Follow-up care was determined by the medical team under whom the patients were admitted and was not influenced by the study. Care often included being followed up by their general practitioner and receiving a respiratory specialist clinic appointment within the 4-month period from discharge. The control group received Smartinhalers to record their adherence, delivered by the physiotherapists to their homes approximately 1 week after discharge.

### Sample Size

It is important to note that this was a pilot trial. However, we calculated a sample size using G power with power set at .90, alpha of .05, and a large effect size *d*=1.15, based on previous research on COPD case management [24]. To find a similar effect size, the required sample size would be 16 patients per group, but 30 patients per group were recruited to allow for inclusion of potential confounding factors in analyses and potential study dropout.

### Primary Outcome

The number of days of hospitalization over the study period for respiratory-related reasons was assessed from hospital records.

### Secondary Outcomes

Adherence to medication was assessed using Adherium Smartinhalers, which electronically recorded inhaler use over the duration of the study from the website portal. Adherence was calculated as a percentage of taken prescribed doses over the 4-month period. Adherence was also measured using the 6-item Medication Adherence Report Scale [25] at baseline and follow-up. Adherence to respiratory exercise was assessed by asking patients how many times they performed their respiratory exercises over the past week at baseline and at follow-up.

Quality of life (consisting of symptoms, functional status, and mental state) was assessed using the CCQ at baseline and follow-up [23]. Hospitalization costs for each participant over the 4-month study period were obtained from hospital records for benefit-cost analyses. Questionnaires at baseline and follow-up were paper based (not Web based) and delivered in person. The follow-up questionnaires were administered at the participant’s home for both groups 4 months after recruitment. There were no changes to trial outcomes after the trial commenced.

### Benefit-Cost Analysis

A benefit-cost analysis was performed by comparing the respiratory-related hospitalization costs per group balanced by the costs of the intervention. It did not include general practitioner costs.

### Process Implementation Interviews

At the end of the study, process implementation interviews were conducted with participants in the intervention group in their homes.

### Data Analysis

Analysis was performed using SPSS (International Business Machines Corporation, United States of America) by a researcher blind to group allocation. Negative binomial logistical regression was conducted to compare the total number of respiratory hospitalizations and days spent in hospital per group. The analysis was repeated when including comorbidities and previous hospital admissions as covariates because these were considerably different between groups at baseline. Intention-to-treat (ITT) and per protocol (PP) analyses are presented for the hospitalization data. The definition of the PP analysis was to include only those patients who received and completed their allocated treatment. Therefore, the 2 people who died and 3 who withdrew before the end of the study period were excluded from the robot group for the PP analysis. Two people who died in the control group and 1 who withdrew were also excluded from the PP analysis. People who received a robot and did not use it much were still included in the PP analysis. Due to the presence of an extreme outlier (46 days in hospital), the hospitalization data were analyzed using bootstrapping. Adherence and CCQ data were analyzed as ITT. The Smartinhaler data were not normally distributed, thus Mann-Whitney *U* tests were performed between groups. For self-reported adherence and CCQ, change scores from baseline to follow-up were calculated, and analyses of covariance were performed controlling for baseline scores and then repeated when also controlling for comorbidities and previous hospitalizations. Spearman correlations were conducted within the intervention group to explore whether the frequency of robot use was associated with medication adherence, rehabilitation exercise, and hospitalizations. The interviews were transcribed and analyzed using an inductive qualitative approach whereby a researcher read the interview transcripts and identified the key themes. Each transcript was then coded for the themes, and themes were checked by another author.

## Results

**Figure 1:**
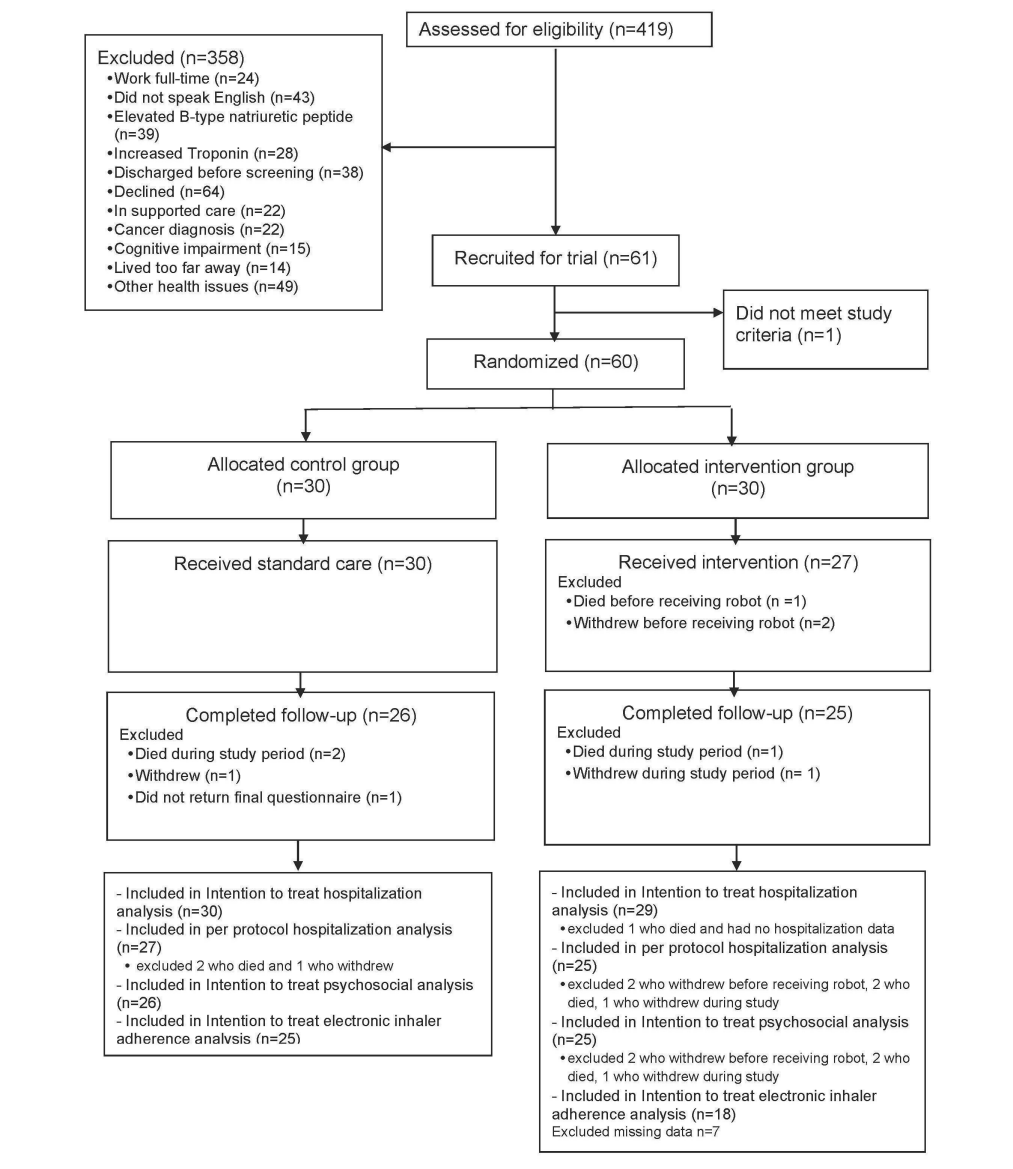
Consolidated Standards of Reporting Trials (CONSORT) flow diagram.

Recruitment was conducted between August 1, 2015 and February 20, 2016 and follow-up between December 1, 2015 and July 1, 2016. Figure 1 shows the participant flow diagram. Sixty patients were randomized, 30 to each group. Robots were delivered to 27 participants in the intervention group (2 dropped out and 1 died before receiving the robots). Twenty-nine were included in the ITT analysis (there was no hospitalization data for the patient who died, but data were available for the dropouts). The PP analysis included 25 in the intervention group because 2 patients withdrew before receiving a robot, 1 withdrew during the study, 1 died before getting the robot, and 1 died during the study period. Twenty-seven were included in the PP control group as 2 died during the study and 1 withdrew. The trial ended when all follow-up data had been collected. There were no unintended effects or harms due to the robots.

Table 1 shows baseline demographic and clinical characteristics. All the participants had at least one other comorbid health problem as assessed by the Charlson Comorbidity Index (mean 2.77 [SD 1.61]), and the intervention group had a trend for more comorbidities. Over the previous 12 months, the average number of hospital admissions for any reason was 1.93 (SD 2.09). On average, patients were admitted for 5.81 days (SD 8.71) over the previous year. Again, there was a trend for patients in the intervention group to have had more days in hospital over the past 12 months than the control group. Of the total number of admissions, the average number due to a respiratory condition was 1.07 (SD 1.69), with an average length of stay of 3.65 days (SD 6.03). Table 1 also shows the medications that patients were on during the study, baseline scores for adherence, and CCQ.

### Primary Outcome

Table 2 shows the total number of respiratory admissions per group and the total number of days spent in hospital for each group in the ITT analysis and the PP analysis. The negative binomial regressions were not significant but were in the intended direction for both the ITT and the PP analyses. When covariates were included in the model, there were still no significant differences between groups, and sensitivity analyses were not significant.

### Secondary Outcomes

ITT analysis showed participants in the intervention group had significantly better adherence to inhaled corticosteroids, long-acting beta agonists, and a combination of these as measured by electronic inhalers (mean 48.5% [SD 34.07]) compared with the control group (mean 29.5% [SD 32.44]; *U*=139.50, *z*=−2.12, *P*=.03, *d*=0.68). When controlling for comorbidities and previous hospital admissions, the results were similar but became nonsignificant (intervention adjusted mean 49.4%, SE=8.23; control adjusted mean 28.9%, SE=6.92; *F*_1,39_=3.44, *P*=.07, partial *η*^2^=0.08; mean difference −20.49, 95% CI −42.84 to 1.84). There was a greater increase in self-reported adherence in the intervention group during the study than in the control group, as shown in Table 3, and this was significant when controlling for covariates.

There were no significant differences between groups in the CCQ using ITT analysis, as indicated in Table 3, although the results were in the intended direction.

There was a significant difference between groups in the self-reported change in the frequency of performing rehabilitation exercises (estimated marginal means control −2.00, intervention 2.54, *F*_1,51_=12.13, *P*=.001, partial *η*^2^=.20, mean difference −4.53, 95% CI −7.16 to −1.92), with the intervention group showing increased frequency of rehabilitation exercises compared with the control group in an ITT analysis.

### Benefit-Cost Analysis

Patient cost data (both direct and total costs for respiratory-related admissions) were extracted from hospital records over the 4-month study period and compared between groups as both ITT and PP (Table 4). Primary care costs were not considered. Multimedia Appendix 1 shows the estimated benefit-cost analysis over 5 years, with the robot showing a net benefit when examining the ITT total cost group comparison. The robot costs reflect the study robot configuration, and in a more commercial product, the robot configuration is likely to be more integrated and less expensive, and thus the long-term benefit-cost may be improved on the analysis shown.

### Process Implementation Interviews

Twenty-five participants from the intervention group were interviewed when the robot was collected. Of those who were not interviewed, 3 patients did not receive the robot at all because of death or because they withdrew from the study before it began, 1 patient died during the study period, and 1 declined to be interviewed.

Nineteen participants thought the robot was useful and had positive comments. Participants liked the robot for a number of reasons, and 4 main themes emerged from their answers (see Table 5). First, all of the participants commented that the robot helped remind them to take their medication (19 participants). A number of participants enjoyed doing the exercises with the robot (12/19 participants). Participants commented that their family and friends were interested in the robot, particularly young children who enjoyed visiting to see the participant as well as the robot. They commented that the robot raised awareness for the family about when the participant should be taking medication and about their illness (14/19 participants). Finally, participants enjoyed having the robot because they felt it had a personality and was good company (15/19 participants). As a consequence, many participants had given their robot a name over the time they had it.

Six participants did not enjoy having the robot. Three of these participants returned the robot before the 4-month period was complete. The negative attitudes toward the robot are presented as themes in Table 5. Participants commented that they did not find the robot useful because they were very good at managing their medication and exercise on their own (5/6 participants), they were unnerved by having the robot in their home (3/6 participants), or they did not like having to manage the robot and could not be bothered with it (all 6 participants). There was a trend for people who disliked the robot to have higher adherence at baseline. This is in line with participants’ comments that they did not find the robot useful because they were already good at managing medication.

### Robot Use

**Figure 2:**
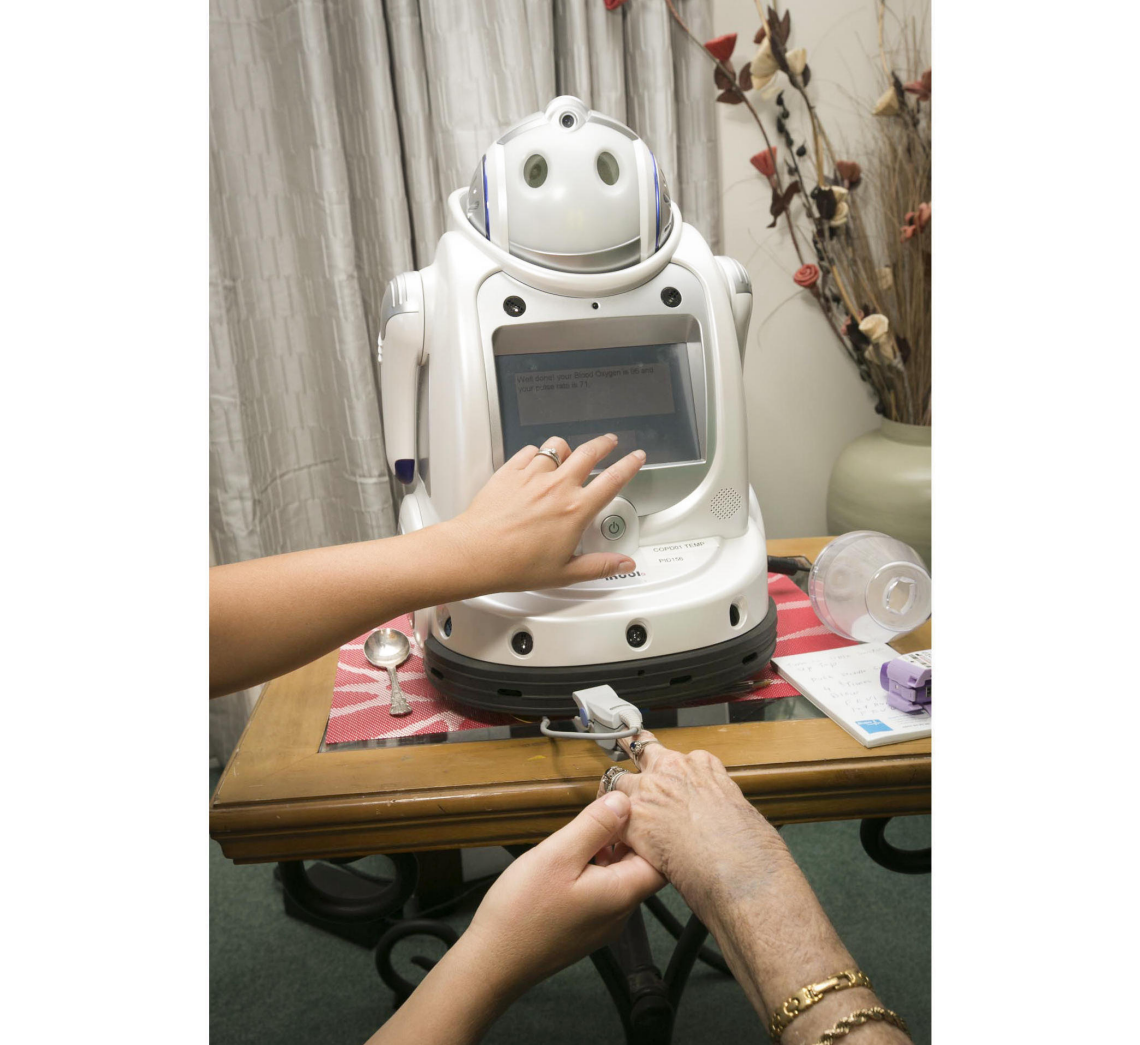
iRobi robot being used by a patient with a physiotherapist showing the functions.

Figure 2 shows the robot being used by a participant with a physiotherapist demonstrating the pulse oximeter function. The function that was used the most was the medication function. Over the 4-month period, it was used on average 464 times per patient (range 41-1509 times). This indicates that, on average, the medication function was used 3 to 4 times a day, which is what would be expected because most patients took medications several times throughout the day. The number of times people used the medication function was significantly associated with better medication adherence measured electronically (Spearman correlation, r_s_=.82, *P*<.001) but not with hospitalizations (r_s_=.26, *P*=.16).

Patients used the exercise function for an average of 84 times over the 4-month period (range 3-221). They used the measurement function for an average of 51 times (range 9-95) and the entertainment function for an average of 29 times (range 1-165). Patients used the education function for an average of 8 times (range 0-77).

**Table 1:** Demographic variables and baseline measures for the control and intervention groups.

Variable			Control (N=30)	Intervention (N=30)	*P* value
Gender—Females, n (%)^a^			18 (60)	19 (63)	.50
Age in years, mean (SD)			69.10 (9.85)	70.57 (10.34)	.58
**Ethnicity, n (%)**^a^			−	−	.56
	NZ^b^ European		16 (52)	15 (50)	−
	Māori		10 (33)	8 (27)	−
	Pacific Island		1 (3)	4 (13)	−
	Other		3 (10)	3 (10)	−
BMI^c^, mean (SD)			27.41 (8.38)	29.16 (8.83)	.46
**Smoking status, n (%)**^a^			−	−	.51
	Current smoker		8 (28)	6 (24)	−
	Ex-smoker		21 (72)	19 (76)	−
**Disease severity at discharge**					
	FEV_1_^d^ percent predicted, mean (SD)		30.27 (12.59)	33.80 (13.61)	.30
	**FEV_1_ severity, n (%)**^a^		−	−	.54
		Moderate	3 (10)	5 (17)	−
		Severe	10 (33)	12 (40)	−
		Very severe	17 (57)	13 (43)	−
SpO_2_^e^, mean (SD)			92.41 (5.28)	93.96 (3.31)	.21
Comorbidities (CCI^f^ total score; mean, [SD])^h^			2.47 (1.76)	3.07 (1.41)	.05
No. of hospital admissions in previous 12 months, mean (SD)^g^			1.47 (1.55)	2.40 (2.46)	.16
No. of days of hospital admissions in past year, mean (SD)^g^			3.83 (5.31)	7.80 (10.87)	.22
No. of hospital respiratory admissions in past year, mean (SD)^g^			0.90 (1.30)	1.23 (2.01)	.67
No. of days of respiratory admissions past year, mean (SD)^g^			2.63 (3.00)	4.67 (7.57)	.49
CCQ^h^ Functional at discharge, mean (SD)			3.75 (1.36)	3.48 (1.19)	.41
CCQ Symptoms at discharge, mean (SD)			3.84 (1.23)	3.60 (1.03)	.41
CCQ Mental at discharge, mean (SD)			3.53 (1.54)	3.88 (1.59)	.96
CCQ Total at discharge, mean (SD)			24.01 (7.12)	23.56 (6.82)	.80
Adherence MARS 5^i^ at discharge^g^, mean (SD)			22.30 (3.76)	21.43 (4.15)	.22
**Medication prescribed during study, n (%)**					
	SABA^j^		23 (77)	26 (87)	.48
	SAMA^k^		2 (7)	4 (13)	.41
	SABA/SAMA combination		2 (7)	1 (3)	.53
	LAMA^l^		22 (73)	24 (80	.71
	LABA^m^		24 (80)	23 (77)	.96
	ICS^n^		4 (13)	4 (13)	.15
	ICS/LABA combination		22 (73)	22 (73)	.84
	Prednisone		17 (57)	18 (60)	.91
	Antibiotic		23 (77)	20 (67)	.30
**Medication assessed for adherence, n (%)**					
	SABA^o^		21 (95)	25 (100)	.47
	SABA/SAMA combination^o^		1 (5)	0 (0)	.47
	ICS^p^		2 (8)	1 (5)	.75
	LABA^p^		1 (4)	0 (0)	.39
	ICS/LABA combination^p^		23 (88)	18 (95)	.46

^a^chi-squared test.

^b^NZ: New Zealand.

^c^BMI: body mass index.

^d^FEV_1_: forced expiratory volume in 1 second.

^e^SpO_2_: pulse oximeter oxygen saturation.

^f^CCI: Charlson Comorbidity Index.

^g^nonparametric test.

^h^CCQ: Clinical COPD Questionnaire.

^i^MARS 5: Medication Adherence Report Scale

^j^SABA: short-acting beta agonists.

^k^SAMA: short-acting muscarinic antagonist.

^l^LAMA: long-acting muscarinic antagonist.

^m^LABA: long-acting beta agonists.

^n^ICS: inhaled steroids.

^o^Control: N=22, Intervention: N=25.

^p^Control: N=26, Intervention: N=19.

**Table 2:** The total number of respiratory admissions per group and the total number of days spent in hospital for each group in the intention to treat (ITT) analysis and the per protocol (PP) analysis, with and without controlling for past year admissions and comorbidities.

Outcome type		Control, (ITT: N=30; PP: N=27)	Intervention, (ITT: N=29; PP: N=25)	Beta	Wald chi-square statistic	95% Wald CI	*P* value
**Intention to treat**							
	Total number of hospitalizations for respiratory problems (ITT), N	15	15	.000	0.000	−0.84 to 0.84	>.99
	Controlling for comorbidities and previous hospitalizations			.129	0.075	−0.80 to 1.06	.79
	Total number of days in hospital for respiratory problems (ITT), N	65	50	.040	0.017	−0.57 to 0.65	.90
	Controlling for comorbidities and previous hospitalizations			.453	1.33	−0.32 to 1.22	.25
**Per protocol**							
	Total number of hospitalizations for respiratory problems (PP), N	15	14	−.008	0.000	−0.92 to 0.90	.99
	Controlling for comorbidities and previous hospitalizations			−.112	−0.044	−1.16 to 1.94	.83
	Total number of days in hospital for respiratory problems (PP), N	67	55	−.120	0.132	−0.53 to 0.77	.72
	Controlling for comorbidities and previous hospitalizations			.370	0.796	−0.44 to 1.18	.37

**Table 3:** Intention-to-treat analyses of difference in secondary outcomes between groups, with and without controlling for covariates.

Outcome		Baseline, mean (SD)		Four months later, mean (SD)		Change score, adjusted mean (SE)		Mean difference (95% CI)	*P* value	Partial eta squared
		Control, n=29	Intervention, n=29	Control, n=27	Intervention, n=25	Control, n=26	Intervention, n=25			
**Adherence**		22.16 (3.76)	21.44 (3.72)	22.27 (4.12)	23.08 (2.63)	0.12 (0.55)	1.63 (0.56)	1.51 (−3.10 to 0.08)	.06	.07
	Additional controls^b^	−	−	−	−	−	−	−1.69 (−3.32 to −0.060	.04	.09
**CCQ**^a^**Functional**		3.74 (1.34)	3.41 (1.18)	2.92 (1.22)	2.32 (1.04)	−0.73 (0.21)	−1.21 (0.21)	0.48 (−0.12 to 1.07)	.11	.05
	Additional controls	−	−	−	−	−	−	0.455 (−0.17 to 1.08)	.15	.04
**CCQ Symptoms**		3.84 (1.20)	3.61 (1.07)	3.08 (1.21)	2.76 (1.01)	−0.61 (0.21)	−.91 (0.22)	0.30 (−0.32 to 0.92)	.33	.02
	Additional controls	−	−	−	−	−	−	0.314 (0.33 to 0.96)	.33	.02
**CCQ Mental**		3.58 (1.50)	3.78 (1.62)	2.33 (1.38)	2.16 (1.43)	−1.25 (0.26)	−1.50 (0.27)	0.26 (0.50 to 1.02)	.50	.01
	Additional controls	−	−	−	−	−	−	0.338 (−0.46 to 1.14)	.34	.02
**CCQ Total**		24.07 (6.90)	23.36 (7.16)	18.17 (6.96)	16.29 (6.30)	−5.46 (1.26)	−7.13 (1.29)	1.67 (−1.95 to 5.29)	.36	.02
	Additional controls	−	−	−	−	−	−	1.82 (−2.04 to 5.68)	.35	.02

^a^CCQ: Clinical COPD Questionnaire.

^b^Controlling for comorbidities and previous hospitalizations.

**Table 4:** Differences in hospitalization costs between groups over the trial period using bootstrapped *t* tests and analysis of covariance with covariates.

Description of cost types		Control, (ITT^a^: N=30; PP^b^: N=27)	Intervention, (ITT: N=29; PP: N=25)	Mean difference (95% CI)	*P* value	Effect size
**Direct costs ITT in NZ**^c^** $, mean (SD)**		2293 (5368)	1140 (2725)	1152 (−760 to 3356)	.32	*d*=0.27
	Controlling for comorbidities and previous hospitalizations	−	−	1173 (−1123 to 3471)	.31	0.02^d^
**Total costs ITT in NZ $, mean (SD)**		3178 (7455)	1599 (3841)	1579 (−1292 to 4451)	.34	*d*=0.27
	Controlling for comorbidities and previous hospitalizations	−	−	1613 (−1587 to 4813)	.32	0.02^d^
**Direct costs PP in NZ $, mean (SD)**		1659 (3633)	1086 (2748)	573 (−1127 to 2436)	.53	*d*=0.18
	Controlling for comorbidities and previous hospitalizations	−	−	497 (−823 to 1929)	.58	0.01^d^
**Total costs PP in NZ $, mean (SD)**		2302 (5079)	1514 (3858)	789 (−1851 to 3192)	.53	*d*=0.17
	Controlling for comorbidities and previous hospitalizations	−	−	686 (−1082 to 2842)	.59	0.01^d^

^a^ITT: intention to treat.

^b^PP: per protocol.

^c^NZ: New Zealand.

^d^Effect size is partial eta squared.

**Table 5:** Positive and negative comments about iRobi from the process implementation interviews.

Comments		Quote
**Positive**		
	Medication reminders	“It made such a difference to my life. I felt that it helped me regain independence and I was breathing better. I was using the preventer regularly and taking my medication.” [Participant 3]
		“It was very helpful at the times I forgot my medication.” [Participant 10]
		“I could rely on it to remind me about my medications, otherwise I would forget.” [Participant 30]
		“Really useful. In the past my ex would text me to tell me to take my medication but the robot was better. I have always been bad at remembering.” [Participant 43]
	Exercise reminders	“It made me aware of when to do my exercises, which was good.” [Participant 5]
		“The reminders about the exercises were good, the robot would tell me to do it and my grandson would come and get me to say the robot needed me.” [Participant 31]
	Family and friends	“If the family are too shy to ask me then they can look on the robot. The robot is not just for me.” [Participant 45]
		“I had a 90 year old friend come over to look at it and she loved it. She wanted one herself.” [Participant 29]
		“Everyone was interested in it when they came over!” [Participant 33]
	Acted like a companion	“I named the robot after my great grandson because I miss him now that he is overseas. It made it like he is here with me.” [Participant 32]
		“Bob (name of the robot) was like one of us. I would pat it on the head and he would respond. I often found myself having conversations with him.” [Participant 38]
		“I will have no friend at home anymore! I liked having it in the house because it talked randomly and I would always touch it as I walked by.” [Participant 58]
**Negative**		
	Not useful	“Not useful. It didn’t do anything for me. I have been doing the same thing for years anyway.” [Participant 25]
		“I didn’t need it. With this illness you never forget to take medication because otherwise you can’t breathe.” [Participant 16]
	Disliked the robot in home	“I felt like I was being policed because people were monitoring how much I was using my inhaler and I felt guilty or like I was being judged. It was an intrusion.” [Participant 14]
		“I felt like my privacy was invaded and I couldn’t go anywhere. I was worried about leaving it at home in case something went wrong or it was stolen.” [Participant 27]
		“The robot would follow me around with its head. I hope that there was not a camera in it.” [Participant 49]
	Found the robot annoying or hard to use	“It drove me batty. It always wanted me to do something.” [Participant 49]
		“I couldn’t read it half the time. I started off doing everything but I had problems going from screen to screen. It got very frustrating. It would tell me to take medications I had already taken.” [Participant 25]

### Technical Issues

Patients in both groups were given the phone number of the physiotherapists to call if there were any issues with the robots and/or Smartinhalers. Patients rang the physiotherapists when they experienced technical issues during the study, including network connection issues, touch screen failures, and hard disk failures on the robot, and the Smartinhalers not charging or connecting correctly. Another issue concerned the pulse oxygen devices, which occasionally became unplugged or had driver issues. In the case of hardware failure, the robot had to be taken away for repair and was replaced with another robot. Technical problems occurred with 50% of the robots delivered.

The time logs kept by the physiotherapists indicated they spent 62 hours in total for troubleshooting technical issues. Calls were mostly from patients in the robot group, as very few patients from the control group contacted the physiotherapists about the Smartinhalers, even if they experienced problems. Most issues could be fixed over the phone, but in a few instances the physiotherapists and/or engineers had to visit the participant’s house. In all cases, the effects of technical failures were negative on patients’ attitudes toward the robots. The physiotherapists spent another 49 hours on patient contact for medical issues that they identified through monitoring (all in the intervention group).

## Discussion

### Principal Findings

This pilot study indicates the feasibility and possible effects of giving a robot to COPD patients at home to help manage their condition. The primary outcome was number of days in hospital, which was not significantly different. This is consistent with recent large-scale studies on telehealth for COPD showing no reductions in hospitalizations [11,12]. The study extends research into the delivery of such care to a robot platform. While the robot did not significantly reduce hospitalizations, many patients appeared to appreciate the robot’s capacity to offer companionship, which may offer benefits over other kinds of platforms such as computers or iPads, although this needs to be further investigated.

The intervention improved adherence to both medication and rehabilitation exercise. Electronic recording showed that adherence to long-acting inhalers was significantly higher in the intervention group. This is backed up by the self-report data showing higher overall medication adherence in the intervention group, which was significantly better than the control group after controlling for past hospitalizations and comorbidities. More frequent use of the medication reminder function on the robot was associated with higher adherence, which suggests the robot was helpful in this regard for at least some patients. Self-reported adherence to rehabilitation exercises significantly increased in the intervention group compared with the control group. It is possible that the social aspects and embodiment of the robot combined with the increased availability of the exercise instructions and the reminders to together create behavior change.

A reduced length of stay might reflect that patients with robots were more comfortable being discharged because of the robot’s presence in the home and the fact that they were being more closely monitored by the physiotherapists. The physiotherapists intervened when they noticed adherence was suboptimal. An illustrative example was when a physiotherapist noticed that the Smartinhaler reliever was used over 30 times in a few minutes, the physiotherapist rang the patient and consulted with a respiratory clinician. The result was a change in medication for the patient and improved management.

About 75% of the sample responded to the robot positively and commented that it helped with medication, education, and companionship. However, 25% of participants did not find the robot useful, and some patients returned it. The patients who did not find it useful tended to have higher adherence at baseline. This suggests that patients should be screened and only offered a robot if they have poor adherence, because this group is the most likely to experience benefits. It also suggests the need to introduce the robot and its functions to the patient when they are an inpatient so that they can make a more informed decision about whether to accept treatment. Anxious patients may find additional benefits from the social presence of the robot, and this is an area for future research.

The robot was programmed to take weekly measurements in the hope of identifying downward trends (which would potentially require a change in management) without placing undue burden on the patient. However, daily measurements are likely better for this purpose. The measurements did serve as a baseline for comparison if patients who contacted us were concerned about a possible exacerbation or deteriorating health.

### Limitations

This study was limited by a small sample, deaths, and dropout, which means the study was underpowered. Our original sample size calculation was optimistic, and a more realistic analysis could have been achieved if it was based on length of hospital stay in COPD patients in the region. The average length of stay in NZ for COPD admissions in 2012 was 4.37 days, and this has been declining over time [7]. For Middlemore hospital over the course of this study, the average length of stay was 4.2 days. The results suggest the intervention had small effects, if any, on hospitalizations, and thus a large sample (similar to recent telehealth trials) would be required to find effects if no changes were made to the robot or protocol in a future study. An additional limitation was that, at baseline, there was a trend that the intervention group had more previous hospitalization days and comorbidities, which could have affected the results. The nonblinding of participants was another limitation, which is common for eHealth trials. The benefit-cost analysis should be viewed with caution because the hospitalization costs were not significantly different between groups—it is provided as an indication only.

The study was conducted in South Auckland, where there is a large population of Māori, Pacific Islanders, and immigrants, and many patients have low socioeconomic status. About a third of the patients were living in rural areas. While these are key groups to target because they tend to have poorer health outcomes, the results may not generalize to all regions. Running the study for a longer period may help indicate whether increased adherence to medication and exercise recommendations could translate into improved quality of life and/or a reduction in hospitalizations.

### Future Work

A number of technical issues would need to be improved before the robot could be implemented on a larger scale, including reliability of the robots and the Internet network. An information technology support company would have to be involved to provide technical support. As a result of this pilot study, the team has now created a software mode to enable the robot to function offline if the network fails intermittently. The software team is working on linking the medication management software with a national electronic prescription database so that medications can be downloaded after the normal entry by pharmacies. In the future, the robot software will need to be integrated with primary care and other Web-based health platforms. Robot studies are expensive, as currently robots must be purchased, programmed, and supported for the purpose of the study. In addition, achieving software reliability is challenging for a study as there is little time for iterative improvement and for establishing a mature software version.

A larger study with more power to detect effects is recommended after improvements to the technology and the trial design are made. It may be useful to screen patients to give the robot only to those who are low in adherence and/or health literacy or who have high levels of anxiety or who are living by themselves. Further work is needed to investigate whether the robot has advantages over a computer tablet providing similar services in this population. The team could possibly program the robot for other long-term conditions such as diabetes and rheumatoid arthritis if given sufficient development time.

The majority of COPD patients admitted to hospital are managed in general medical wards and by a team not involved in their long-term care. Our experience suggests that physiotherapists and respiratory nurse specialists involved in long-term care (eg, pulmonary rehabilitation, outpatient care, outreach or integrated care programs) are ideal people to implement telehealth programs using robots. Many patients recruited into this study appeared to be on suboptimal bronchodilator therapy with insufficient numbers on long-acting muscarinic antagonists (LAMAs) in particular. In NZ, access to LAMAs is through special authority application, and patients can only be prescribed LAMAs if their FEV_1_ is <60% predicted. Spirometry was infrequently recorded on patients during their admission, and <50% of general practitioners in NZ have spirometers. This nonadherence to international guidelines might be one reason as to why NZ has the fourth highest admission rates for COPD in the world.

Ideally, patients would be introduced to the robot while in hospital to gauge whether they like the concept and can adapt to the measurement requirements. They would also benefit from a formal review by a respiratory physician before discharge. Some thought also needs to be given as to how to achieve a 24-hour monitoring of the Web server’s patient information 7 days a week. This would potentially require a national or regional solution that includes a 24/7 monitoring system such as an extension of the New Zealand Healthline, with instant links back to the health care team members when patients are becoming unstable. A similar solution for diabetes and asthma management could be set up, which would reduce costs and pay for infrastructural development.

### Conclusions

A robot may be useful for COPD patients who are struggling with medication adherence and rehabilitation exercises to improve adherence, although hospitalizations may not be reduced. We recommend improvements to the robot, changes to the way it is incorporated into the health care system, and a larger study comparing robots with other forms of technology, before stronger conclusions can be made.

## Appendix

Multimedia Appendix 1 Benefit-cost analysis based on intention to treat analysis of mean total costs per patient in each group.

Multimedia Appendix 2 CONSORT‐EHEALTH checklist (V.1.6.1).

## Abbreviations

BMI: body mass index
CCI: Charlson Comorbidity Index
CCQ: Clinical Chronic Obstructive Pulmonary Disease Questionnaire
COPD: chronic obstructive pulmonary disease
CURB-65: Confusion, bUn>19mg/dL, RR>30, BP<90 SBP or <60 DBP, Age>65
FEV1: forced expiratory volume in 1 second
ICS: inhaled corticosteroids
ITT: intention to treat
LABA: long-acting beta agonists
LAMA: long-acting muscarinic antagonist
NT-pro BNP: N-terminal pro b-type natriuretic peptide
NZ: New Zealand
PP: per protocol
SABA: short-acting beta agonists
SAMA: short-acting muscarinic antagonist
SMS: short message service
SpO_2_: peripheral capillary oxygen saturation
