# Supplementary material for: Using Robots at Home to Support Patients With Chronic Obstructive Pulmonary Disease: Pilot Randomized Controlled Trial
Source: J Med Internet Res. 2018 Feb 13;20(2):e45. doi: 10.2196/jmir.8640 (PMC5829456; doi:10.2196/jmir.8640)
Supplement: Multimedia Appendix 1 [file jmir_v20i2e45_app1.pdf]

			Patients	4 months	Annualized
**Benefit:**					
	Mean difference in total hospital costs per patient	NZ$1579	30	NZ$47,370	NZ$142,110
**Costs Ongoing:**					
	Phone communication plans NZ$46 per patient for 4 months	NZ$184	30	NZ$5520	NZ$16,560
	Wi-Fi plans per robot NZ$16 per month per patient for 4 months	NZ$64	30	NZ$1920	NZ$5760
	Physiotherapist time 238 hours *NZ$40 per hour	NZ$40	238	NZ$9520	NZ$28,560
	Total			NZ$16,960	NZ$50,880
**Net benefit per annum**				NZ$30,410	**NZ$91,230**
Present Value of annual benefits in real terms @ 3% real required rate of return					NZ$417,807

**Investment Cost**		
Robot purchase	NZ$90,000	
Dongles	NZ$2970	
Phones	NZ$27,000	
Initial training	NZ$240	NZ$120,210
**Net present value**		**NZ$297,597**

**Return on Investment:**							
Annual benefit							NZ$91,230
Annual Expenses:		***Number:***	***Cost:***	***Total Cost:***	***Write-off Years:***	***Annual Depreciation:***	
Depreciation:	Robots	30	NZ$3000	NZ$90,000	5	NZ$18,000	
	Dongles	30	NZ$99	NZ$2970	5	NZ$594	
	Phones	30	NZ$900	NZ$27,000	5	NZ$5400	
	Training						
Amortization:	Hours	6	NZ$40	NZ$240	5	NZ$48	
							NZ$24,042
Annual Profit							**NZ$67,188**
Average Investment Cost over 5 years							**NZ$60,105**
**Return on Investment**							**112%**
